# Five-factor personality traits in patients with schizophrenia and bipolar disorder: a systematic review and meta-analysis

**DOI:** 10.1093/ijnp/pyaf060

**Published:** 2025-08-08

**Authors:** Mayuka Hashimoto, Kazutaka Ohi, Daisuke Fujikane, Kentaro Takai, Ayumi Kuramitsu, Yukimasa Muto, Shunsuke Sugiyama, Toshiki Shioiri

**Affiliations:** Department of Psychiatry, Gifu University Graduate School of Medicine, Gifu, Japan; Japanese Red Cross Gifu Hospital, Gifu, Japan; Department of Psychiatry, Gifu University Graduate School of Medicine, Gifu, Japan; Department of Psychiatry, Gifu University Graduate School of Medicine, Gifu, Japan; Department of Psychiatry, Gifu University Graduate School of Medicine, Gifu, Japan; Department of Psychiatry, Gifu University Graduate School of Medicine, Gifu, Japan; Department of Psychiatry, Gifu University Graduate School of Medicine, Gifu, Japan; Department of Psychiatry, Gifu University Graduate School of Medicine, Gifu, Japan; Department of Psychiatry, Gifu University Graduate School of Medicine, Gifu, Japan

**Keywords:** schizophrenia, bipolar disorder, NEO five-factor inventory, extraversion, openness, agreeableness

## Abstract

**Background:**

Personality traits play crucial roles in the onset, manifestation, and course of schizophrenia and bipolar disorder (BD). Previous meta-analyses focusing on NEO personality traits in patients with schizophrenia and BD revealed distinct differences in specific personality traits between patients with schizophrenia and healthy controls and between patients with BD and healthy controls. However, direct comparisons of personality profiles between schizophrenia patients and BD patients have been limited, with existing studies often limited by relatively small sample sizes.

**Methods:**

Two online databases (PubMed and Scopus) were searched systematically to identify relevant articles, including publications up to April 2024. A meta-analysis of five personality traits, namely, neuroticism (N), extraversion (E), openness (O), agreeableness (A), and conscientiousness (C), assessed by the NEO five-factor inventory, was performed in seven cohorts, including our patient samples, consisting of 768 patients with schizophrenia and 555 patients with BD.

**Results:**

There was no significant heterogeneity in the five personality traits among the seven studies (*I^2^* = 0-53.8, *P* > .05), except for C (*I^2^* = 77.1, *P* = 5.65 × 10^-4^). Our meta-analyses revealed significant differences in three personality traits (E, O, and A) between patients with schizophrenia and patients with BD (E: Hedges’ *g* = −0.40, *P* = 1.34 × 10−^11^; O: *g* = −0.22, *P* = 1.76 × 10−^4^; and A: *g* = −0.24, *P* = 3.73 × 10^-5^). Patients with schizophrenia had lower scores on E, O, and A than those with BD did. No significant differences in the other two traits, N and C, were observed between the groups (*P* > .05).

**Conclusions:**

Our findings suggest that schizophrenia patients and BD patients have distinct personality profiles and that schizophrenia patients have more pronounced personality profiles than BD do, despite their overlapping symptoms and genetic predispositions.

Significance Statement We compared the NEO five personality traits—N, E, O, A, and C—between 768 patients with schizophrenia (SCZ) and 555 patients with BD. SCZ had lower scores on E, O, and A than BD. These effect sizes were modest: E (*g* = −0.40), O (*g* = −0.22), and A (*g* = −0.24), which were intermediate between personality traits in SCZ compared with controls and personality traits in BD compared with controls. SCZ have more pronounced personality profiles in terms of E, O and A than BD.

## INTRODUCTION

Schizophrenia and bipolar disorder are common psychiatric disorders, each affecting approximately 1% of the population over a lifetime and impacting individuals and their families. While distinct in their diagnostic criteria—schizophrenia is characterized primarily by positive and negative symptoms and difficulties in cognitive and social functioning, whereas bipolar disorder is characterized by severe mood swings, including periods of mania and depression—there are both clinical and underlying genetic similarities and differences between these disorders.^1–6^

Personality traits play crucial roles in the onset, manifestation, and course of schizophrenia and bipolar disorder.^7–9^ The relationships between these disorders and personality traits provide an intriguing perspective for understanding their pathogenesis and progression. Specifically, certain personality profiles—particularly high levels of neuroticism (N) and low levels of extraversion (E)—not only predispose individuals to a greater risk of developing these disorders^8^^,^^10^ but also influence the severity of their symptoms, low tolerance for stress and anxiety, and cognitive and social functioning.^10–12^ Indeed, these personality features may serve as both precursors and perpetuators of the disorders,^13^ potentially guiding their trajectory and influencing the effectiveness of therapeutic interventions.

The five-factor model (FFM), or the Big Five personality traits—N, E, openness (O), agreeableness (A), and conscientiousness (C)—has become prevalent in psychiatric research.^14^ This model provides a comprehensive framework for examining personality dimensions that remain remarkably consistent across various cultures, languages, and age groups. The universality of the FFM supports its utility in investigating the relationships between personality and psychiatric disorders across diverse populations.^15–17^

Previous meta-analyses focusing on NEO personality traits between patients with schizophrenia and those with bipolar disorder and healthy controls (HCs) have revealed significant differences in specific personality traits between these case–control groups. Compared with HCs, patients with schizophrenia tend to exhibit higher levels of N and lower levels of E, O, A, and C (Figure 3A), with these differences often demonstrating moderate to large effect sizes that are not influenced by demographic factors such as age or sex.^18^ In contrast, individuals with bipolar disorder present with higher N and lower E and C than do HCs (Figure 3B), indicating a distinct personality profile that may affect the onset and progression of the disorder.^19^ However, direct comparisons of personality profiles between patients with schizophrenia and those with bipolar disorder have been limited, with existing studies often limited by relatively small sample sizes.^20–23^ These findings suggest a need for more comprehensive investigations to understand the differences in personality traits between these two disorders. We hypothesized that schizophrenia and bipolar disorder exhibit distinct personality profiles, despite their overlapping symptoms and genetic predispositions.

In this study, we performed a meta-analysis of personality traits in patients with schizophrenia and bipolar disorder in large cohorts, including our patient samples (*n* = 7) of 768 patients with schizophrenia and 555 patients with bipolar disorder. Using the FFM, we compared the five personality profiles, N, E, O, A, and C, between these groups. Through this comprehensive approach, we sought to contribute to the understanding of how these personality traits may distinguish between these two psychiatric disorders.

## METHODS

### Our Japanese Case Cohorts

The subjects in the Japanese case cohort consisted of 182 patients with schizophrenia [46.7% males (85/97); mean age ± S.D., 47.1 ± 13.5 years] and 26 patients with bipolar disorder [42.3% males (11/15); 50.5 ± 17.5 years] in Kanazawa, Japan, and 53 patients with schizophrenia [35.9% males (19/34); 40.5 ± 17.4 years] and 14 patients with bipolar disorder [28.6% males (4/10); 53.9 ± 16.8 years] in Gifu, Japan (Table S1). Patients with schizophrenia in Gifu were younger than those with bipolar disorder in Gifu were (*z* = 2.5, *P* = .011), whereas patients with schizophrenia in Kanazawa had fewer years of education than those with bipolar disorder in Kanazawa did (*z* = 2.2, *P* = .03) (Table S1). All the subjects were biologically unrelated and of Japanese ethnicity and were recruited from among both outpatients and inpatients at Kanazawa Medical University and Gifu University hospitals and related hospitals in Japan. The patients were recruited from the Schizophrenia Non-Affected Relative Project.^4^^,^^24–27^ A detailed description of patient recruitment and diagnosis has been provided previously.^24–26^ Briefly, each patient with schizophrenia and bipolar disorder was diagnosed based on unstructured clinical interviews, medical records, and clinical conferences according to the criteria in the fifth edition of the *Diagnostic and Statistical Manual of Mental Disorders* (DSM-5). Patients with comorbid substance-related disorders or intellectual disability were excluded. Subjects were excluded from this analysis if they had neurological or medical conditions that could affect the central nervous system, such as atypical headaches, head trauma with loss of consciousness, chronic lung disease, kidney disease, liver disease, thyroid disease, active cancer, cerebrovascular disease, epilepsy or seizures. Current clinical symptoms were evaluated via the Positive and Negative Syndrome Scale,^28^ the 17-item Hamilton Rating Scale for Depression^29^ and the Young Mania Rating Scale.^30^ A written informed consent was obtained from all the subjects after the procedures were fully explained. This study was carried out in accordance with the World Medical Association’s Declaration of Helsinki and approved by the Research Ethics Committees of Kanazawa Medical University and Gifu University.

### Five-Factor Personality Traits

To measure five-factor personality traits, the NEO-FFI, which is a well-established self-report questionnaire,^14^ was used. The NEO-FFI is a shortened version of the NEO-PI-R consisting of 60 questions. We examined the main scores for five traits (N, E, O, A, and C) of the scale. The concepts of each trait are as follows: N is the tendency toward negative emotion and apprehension. E is engagement with the external world and assertiveness. O is appreciation for variety and wanderlust. A is concerned with social harmony and politeness. C is the temperament of self-discipline and orderliness.

### Meta-Analysis of Studies Using NEO

Before studies were searched for meta-analyses, registration and protocols were not registered or prepared. We first searched for the studies that we used for our meta-analysis in the PubMed and Scopus databases with the search terms (“NEO” or “five-factor” or “five factor” or “NEO-FFI” or “NEO-PI-R” or “Big five” or “NEO Five-Factor Inventory” or “Basic personality traits” or “Neuroticism” or “Extraversion” or “Openness” or “Agreeableness” or “Conscientiousness”) and (“schizophrenia” or “schizophr^*^”) and (“bipolar”). Our search data encompassed all publications up to April 2024. Additionally, references cited in the publications that we obtained were searched to identify additional potentially relevant studies that might not be listed in PubMed or Scopus. Next, studies were included in the meta-analysis if they met the following criteria: (1) published in a peer-reviewed journal in English; (2) compared patients with schizophrenia to patients with bipolar disorder using the NEO-FFI, NEO-PI-R, or NEO-related inventory; and (3) contained information on the means and standard deviations (SDs) or medians and median absolute deviations for each personality trait for both patients with schizophrenia and patients with bipolar disorder. The available information on age, sex, age at onset, duration of illness and diagnostic criteria was also collected. Two raters (MH and KO) independently verified the validity of the data in all of the included articles.

### Statistical Analyses

The meta-analyses were performed via the Comprehensive Meta-analysis Version 2.0 software package.^31^ Cochran’s Q test was performed to assess possible heterogeneity between the individual studies. The effect sizes and 95%CIs were estimated under the random-effects model described by DerSimonian and Laird if there was evidence of heterogeneity (*P*(*Q*) < 0.05). Otherwise, the fixed-effects model described by Mantel–Haenszel was used (*P*(*Q*) ≥ 0.05). Publication bias was assessed via Egger’s regression asymmetry test with a funnel plot of the effect size against standard error in each study.^32^ Effect sizes (Hedges’ *g*) indexing the standardized difference in each score between patients with schizophrenia and patients with bipolar disorder were calculated on the basis of reported statistics (the mean of the schizophrenia patients minus the mean of the bipolar disorder patients, divided by the pooled SD).^33^ Effect sizes are typically categorized as small (*g* = 0.2), medium (*g* = 0.5) or large (*g* = 0.8). To control for differences in sample size between studies when mean effect sizes were computed, studies were weighted according to estimates of inverse variance. To determine whether the mean effect sizes were statistically significant, the CI and z transformation of the effect size were used. The effect sizes and 95% CIs are represented by forest plots. A leave-one-out sensitivity analysis was performed by iteratively removing one study at a time to confirm that our findings were not driven by any single study. The significance level was set as two-tailed *P* < .05 for all the statistical tests. The significance level was adjusted via the Bonferroni correction for five independent traits (*P* <. 01, *α* = 0.05/5 traits).

## RESULTS

### Selection Process and Characteristics of Study Cohorts for Meta-Analysis of Schizophrenia and Bipolar Disorder

**Figure 1:**
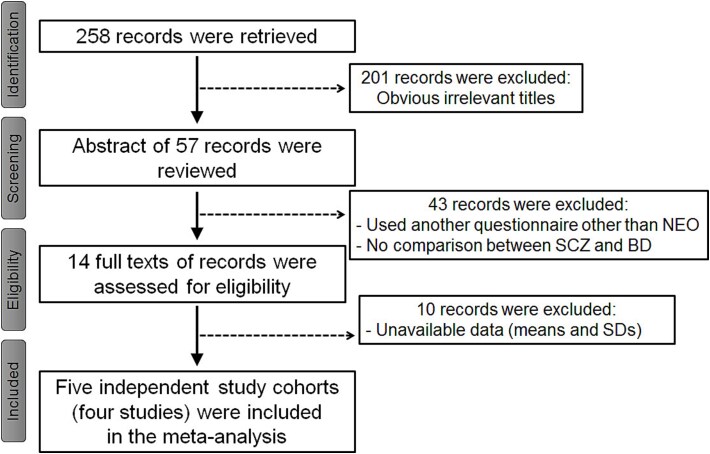
Flow chart of the literature search for meta-analyses. SCZ, schizophrenia; BD, bipolar disorder.

We identified 258 relevant articles in PubMed and Scopus via the search terms mentioned in the Methods section. A total of five independent study cohorts (four studies)^20–23^ and our two cohorts (Kanazawa and Gifu, Japan) met the inclusion criteria for our meta-analysis (with a total of 768 schizophrenia patients and 555 bipolar disorder patients) (Figure 1). Kalman et al. (2022) used two different subject cohorts (Munich, Germany, and multiple sites, Germany/Austria). Heerlein et al. (1996) assessed only N and E using the Munich Personality Test. All participants included in each study were assessed via the NEO-FFI, the NEO-PI-R or the Big Five Inventory. The characteristics of the participants included in our meta-analyses are shown in Table 1 and Table S1.

**Table 1:** Demographic information included in the meta-analyses of NEO five-factor personality traits

	**Country**	**Schizophrenia (*n* = 768)**					**Bipolar disorder (*n* = 555)**					**Diagnostic**
**Authors**	**(City)**	***n***	**Age**	**%M**	**AAO**	**DOI**	***n***	**Age**	**%M**	**AAO**	**DOI**	**criteria**
**Bagby et al. (1997)**	Canada (Toronto)	41	40.3 ± 8.1	61.0	21.1 ± 4.3	NA	34	37.7 ± 10.5	26.5	25.8 ± 8.4	NA	DSM-III-R
**Furukawa et al. (1998)**	Japan (Nagoya)	19	NA	NA	NA	NA	8	NA	NA	NA	NA	ICD-10
**Heerlein et al. (1996)**	Chile (Chile)	16	27.6 ± 9.6	81.3	NA	NA	21	34.0 ± 15.3	61.9	NA	NA	DSM-III-R, ICD-10
**Kalman et al. (2022)^a^**	Germany (Munich)	92	38.5 ± 11.1	62.4	NA	15.0 ± 11.9	123	42.6 ± 13.0	48.8	NA	14.0 ± 11.9	NA
**Kalman et al. (2022)^b^**	Germany/Austria (multisites)	365	40.4 ± 12.0	65.0	NA	13.0 ± 11.9	329	45.6 ± 13.0	50.4	NA	13.0 ± 11.9	DSM-IV
**Present study^a^**	Japan (Kanazawa)	182	47.1 ± 13.5	46.7	26.9 ± 10.2	20.2 ± 13.1	26	50.5 ± 17.5	42.3	35.0 ± 16.8	15.5 ± 12.7	DSM-5
**Present study^b^**	Japan (Gifu)	53	40.5 ± 17.4	35.9	29.2 ± 13.6	11.3 ± 11.0	14	53.9 ± 16.2	28.6	35.1 ± 15.9	18.7 ± 9.7	DSM-5

%M, %Male; AAO, age at onset (years); DOI, duration of the illness (years); NA, not applicable. The means ± SDs are shown. Kalman et al. (2022) reported the medians and median absolute deviations of age and DOI.

### Meta-Analyses to Estimate the Differences in Personality Traits between Patients with Schizophrenia and Patients with Bipolar Disorder

**Figure 2:**
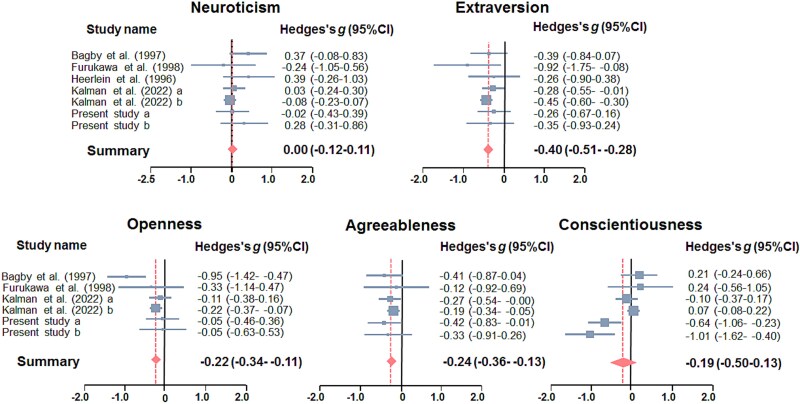
Differences in five personality traits between patients with schizophrenia (SCZ) and those with bipolar disorder (BD). The effect sizes (Hedges’ *g*) with 95% CIs for each study are presented in the forest plots. The diamond in the bottom portion represents the pooled effect size with a 95% CI. A positive effect size means that SCZ patients have higher scores than BD patients do, whereas a negative effect size means that SCZ patients have lower scores than BD patients do.

**Figure 3:**
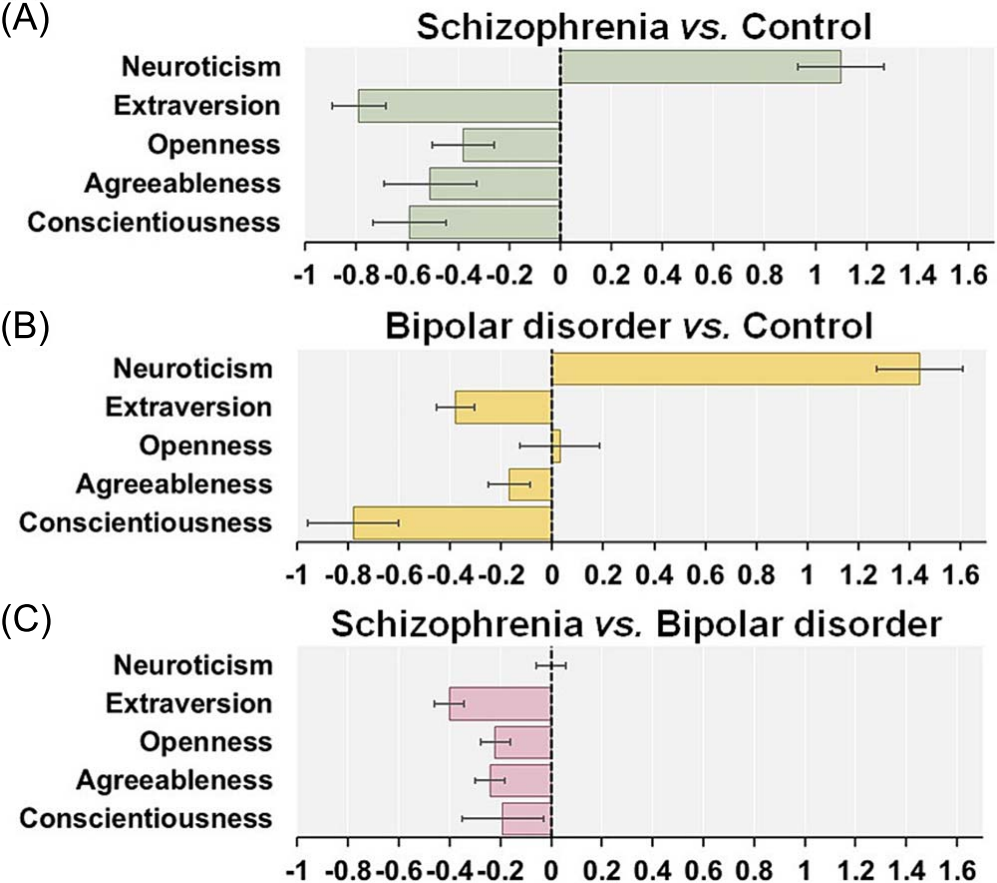
Degree of differences in personality traits between patients with schizophrenia and controls (**A**) (Ohi et al., 2016), between patients with bipolar disorder and controls (**B**) (Hanke et al., 2022), and between patients with schizophrenia and those with bipolar disorder (**C**) (present study). Effect sizes (Hedges’ *g*) with standard errors for each personality trait between groups are presented.

For the five personality traits, there was significant heterogeneity in C among the studies (*I^2^* = 77.1, *P* = 5.65 × 10^-4^), and a random effects model was used for the meta-analysis. In contrast, there was no significant heterogeneity in other personality traits among the studies (*P* > .05), and the fixed-effects model was applied for other traits. According to Begg’s funnel plot test for asymmetry (Figure S1), no evidence of publication bias was detected for any personality traits (*P* > .05). Our meta-analyses revealed significant differences in three personality traits (E, O, and A) between patients with schizophrenia and patients with bipolar disorder (Figures 2 and 3C and E: Hedges’ *g* = −0.40, *P* = 1.34 × 10^-11^; O: *g* = −0.22, *P* = 1.76 × 10^-4^; A: *g* = −0.24, *P* = 3.73 × 10^-5^). Patients with bipolar disorder had higher scores on E, O, and A than did those with schizophrenia. No significant differences in the other two traits (N and C) were observed between the groups (*P* > .05).

To assess whether any individual study has a biased impact on our outcomes, we further performed “leave-one-out” sensitivity analyses for each personality trait (Table 2). The overall effect size and *p* values were recalculated when each study was removed. Even after sensitivity analyses were performed, there was still significant heterogeneity in C among the studies (*I^2^* > 66.0, *P*(Q) < 0.05). Furthermore, sensitivity analysis for the O trait demonstrated significant heterogeneity among studies (*I^2^* > 59.5, *P*(Q) < 0.05) when each study was removed, except for Bagby’s study (1997) (*I^2^* = 0, *P*(Q) = 0.87), and the effect size for O decreased when Bagby’s study (1997) was removed (*g* = −0.18, *P* = 4.00 × 10^-3^). In contrast, the sensitivity analyses revealed that there was no significant heterogeneity in other personality traits, N, E, or A, among the studies (*P*(Q) > 0.05), and there were significant differences in the E and A traits between patients with schizophrenia and patients with bipolar disorder (Table 2, E: −0.42 > *g* > -0.33, 1.67 × 10^-11^ < *P* <3.41 × 10^-4^, A: −0.32 > *g* > −0.23, 3.70 × 10^-5^ < *P* < 6.45 × 10^-4^).

**Table 2:** Leave-one-out sensitivity analyses for the meta-analyses of personality traits

	*N*	SCZ	BD	*P* (*Q*)	*I^2^*	Hedges’*g* (95% CI)	*P* (*Z*)
**Neuroticism (N)**							
** Overall**	7	768	555	0.39	5.1	0.00 (−0.12−0.11)	0.98
** Bagby et al. (1997)**	6	727	521	0.62	0	−0.03 (−0.14−0.09)	0.66
** Furukawa et al. (1998)**	6	749	547	0.31	16.3	0.00 (−0.11−0.12)	0.95
** Heerlein et al. (1996)**	6	752	534	0.43	0	−0.01 (−0.13−0.10)	0.82
** Kalman et al. (2022)^a^**	6	676	432	0.28	20.2	−0.01 (−0.13−0.12)	0.91
** Kalman et al. (2022)^b^**	6	403	226	0.58	0	0.11 (−0.07−0.29)	0.23
** Present study^a^**	6	586	529	0.28	20.8	0.00 (−0.12−0.12)	0.99
** Present Study^b^**	6	715	541	0.37	7.9	−0.01 (−0.13−0.10)	0.84
**Extraversion (E)**							
** Overall**	7	768	555	0.77	0	−0.40 (−0.51−0.28)	**1.34 × 10** ^−**11**^
** Bagby et al. (1997)**	6	727	521	0.66	0	−0.40 (−0.52−0.28)	**5.54 × 10** ^−**11**^
** Furukawa et al. (1998)**	6	749	547	0.88	0	−0.39 (−0.50−0.27)	**6.51 × 10** ^−**11**^
** Heerlein et al. (1996)**	6	752	534	0.69	0	−0.40 (−0.52−0.29)	**1.67 × 10** ^−**11**^
** Kalman et al. (2022)^a^**	6	676	432	0.79	0	−0.42 (−0.55−0.30)	**7.50 × 10** ^−**11**^
** Kalman et al. (2022)^b^**	6	403	226	0.81	0	−0.33 (−0.51−0.15)	**3.41 × 10** ^−**4**^
** Present study^a^**	6	586	529	0.74	0	−0.41 (−0.53−0.29)	**2.20 × 10** ^−**11**^
** Present Study^b^**	6	715	541	0.66	0	−0.40 (−0.52−0.28)	**2.65 × 10** ^**-11**^
**Openness (O)**							
** Overall**	6	752	534	0.055	53.8	−0.22 (−0.34−0.11)	**1.76 × 10** ^**−4**^
** Bagby et al. (1997)**	5	711	500	0.87	0	−0.18 (−0.30−0.06)	**4.00 × 10** ^**−3**^
** Furukawa et al. (1998)**	5	733	526	**0.030**	62.6	−0.25 (−0.49−0.01)	**0.042**
** Kalman et al. (2022)^a^**	5	660	411	**0.043**	59.5	−0.31 (−0.60−0.01)	**0.043**
** Kalman et al. (2022)^b^**	5	387	205	**0.029**	62.8	−0.28 (−0.62−0.06)	0.10
** Present study^a^**	5	570	508	**0.040**	60.2	−0.30 (−0.56−0.04)	**0.025**
** Present study^b^**	5	699	520	**0.034**	61.6	−0.28 (−0.53−0.03)	**0.026**
**Agreeableness (A)**							
** Overall**	6	752	534	0.86	0	−0.24 (−0.36−0.13)	**3.73 × 10** ^**−5**^
** Bagby et al. (1997)**	5	711	500	0.86	0	−0.23 (−0.35−0.11)	**1.48 × 10** ^**−4**^
** Furukawa et al. (1998)**	5	733	526	0.77	0	−0.25 (−0.37−0.13)	**3.70 × 10** ^**−5**^
** Kalman et al. (2022)^a^**	5	660	411	0.77	0	−0.24 (−0.37−0.11)	**2.91 × 10** ^**−4**^
** Kalman et al. (2022)^b^**	5	387	205	0.94	0	−0.32 (−0.51−0.14)	**6.45 × 10** ^**−4**^
** Present study^a^**	5	570	508	0.89	0	-0.23 (−0.35−0.11)	**2.10 × 10** ^**−4**^
** Present study^b^**	5	699	520	0.77	0	−0.24 (−0.36−0.12)	**6.73 × 10** ^**−5**^
**Conscientiousness (C)**							
** Overall**	6	752	534	**5.65 × 10** ^**−4**^	77.1	−0.19 (−0.50−0.13)	0.24
** Bagby et al. (1997)**	5	711	500	**3.99 × 10** ^**−4**^	80.5	−0.27 (−0.63−0.09)	0.14
** Furukawa et al. (1998)**	5	733	526	**2.73 × 10** ^**−4**^	81.2	−0.23 (−0.57−0.10)	0.17
** Kalman et al. (2022)^a^**	5	660	411	**2.34 × 10** ^**−4**^	81.5	−0.22 (−0.65−0.21)	0.32
** Kalman et al. (2022)^b^**	5	387	205	**2.84 × 10** ^**−3**^	75.2	−0.27 (−0.68−0.15)	0.21
** Present study^a^**	5	570	508	**0.011**	69.5	−0.08 (−0.33−0.22)	0.59
** Present study^b^**	5	699	520	**0.019**	66.0	−0.07 (−1.62−0.20)	0.62

*N*, number of studies; SCZ, schizophrenia; BD, bipolar disorder. *P*(*Q*): Cochran’s *Q* test was used to assess the heterogeneity among studies. *P*(*Z*): The *z* test was used to determine the significance of the overall effect size when SCZ patients were compared with BD patients. *P* values <.05 are shown in boldface. ^a,b^Refer to two distinct cohorts within the same study.

## DISCUSSION

This is the first pooled study comparing the NEO five personality traits—N, E, O, A, and C—between 768 patients with schizophrenia and 555 patients with bipolar disorder. There was no significant heterogeneity in personality traits, except for C, among the seven studies. Patients with schizophrenia had significantly lower scores on E, O, and A than did those with bipolar disorder. The differences in E and A between disorders were consistent across studies, whereas the difference in O between disorders was influenced mainly by Bagby’s study. Our findings suggest that schizophrenia and bipolar disorder patients have distinct personality profiles, despite their overlapping symptoms and genetic predispositions.

We revealed that schizophrenia patients had lower levels of E (*g* = −0.40), O (*g* = −0.22) and A (*g* = −0.24) than bipolar disorder patients did. As shown in Figure 3, previous meta-analyses of the NEO five personality traits between patients with schizophrenia and controls indicated that patients with schizophrenia had lower levels of E (*g* = −0.79), O (*g* = −0.38) and A (*g* = −0.51) than controls did (Figure 3A).^18^ Moreover, previous meta-analyses of personality traits between patients with bipolar disorder and controls demonstrated that bipolar disorder patients had lower levels of E (*g* = −0.38) than controls did, whereas no significant differences in O (*g* = 0.03) or A (*g* = −0.17) were observed between bipolar disorder patients and controls (Figure 3B).^19^ These findings highlight that patients with schizophrenia have more pronounced personality profiles in terms of E, O and A relative to not only controls but also patients with bipolar disorder.

The reasons for low E, O and A in schizophrenia patients might be related to the symptoms of schizophrenia and the social context. E, a personality trait characterized by sociability, assertiveness, and positive emotions, has been shown to inversely correlate with negative symptoms in patients with schizophrenia.^12^^,^^34^^,^^35^ Consistent with these studies, our Japanese patients with schizophrenia also displayed a negative partial correlation between E and negative symptoms, with age, sex and institute as covariates (*r* = −0.14, *P* = .032). Furthermore, lower E was associated with a reduced volume of the fusiform gyrus, which is involved in facial discrimination, in patients with schizophrenia.^36^ These findings indicate that schizophrenia patients with lower levels of E tend to exhibit more severe negative symptoms and impaired facial discrimination, leading to poorer social functioning.

O represents how receptive a person is to new experiences and ideas, reflecting their creativity, artistic sensitivity, and intellectual curiosity. A meta-analysis indicated that patients with schizophrenia were less creative, which represents O, than control subjects were.^37^^,^^38^ In particular, the study revealed that the difference in creativity was greater in chronic patients and inpatients than in acute and early-onset patients and outpatients.^38^ Furthermore, creativity was negatively correlated with negative symptoms.^38^ A study reported that O was negatively correlated with negative symptoms,^39^ although there was a nominal partial correlation between O and negative symptoms in our Japanese patients with schizophrenia (*r* = −0.12, *P* = .07). On the other hand, a positive correlation between fewer years of education and lower O has been shown in patients with schizophrenia.^20^ A similar marginally positive partial correlation between O and years of education was observed in our Japanese patients with schizophrenia (*r* = 0.11, *P* = .08). Indeed, several studies have indicated that years of education in patients with schizophrenia are significantly lower than those in patients with unipolar and bipolar disorders.^6^^,^^20^ Therefore, these findings suggest that decreased O might represent a secondary outcome related to increased negative symptoms and/or decreased educational achievements in patients with schizophrenia.

A assesses traits related to an individual’s attitudes and behavior toward others and is a component that measures empathy, friendliness, tolerance, trustworthiness and cooperativeness toward others. Negative correlations between A and psychotic symptoms have been demonstrated in patients with schizophrenia.^12^^,^^35^^,^^40^ Severe positive as well as negative symptoms were correlated with lower A in these patients. Consistent with these previous studies,^12^^,^^40^ our Japanese patients with schizophrenia also displayed a negative partial correlation between A and positive symptoms, with age, sex, and institute as covariates (*r* = −0.202, *P* = .002). Emotional recognition deficits in patients with schizophrenia are strongly correlated with both positive and negative symptoms.^41^^,^^42^ Furthermore, insular hyperactivity of responses to facial expressions of disgust was correlated with lower A in patients with schizophrenia.^43^ Thus, the enhanced insular cortical response to expressions of disgust may be a neural marker of A. Therefore, these findings suggest that insular hyperactivity of responses might cause emotional recognition deficits and psychotic symptoms, resulting in decreased A.

As shown in Table 2, high heterogeneity in C remained even after leave-one-out sensitivity analyses (*I^2^* > 66.0% in all iterations), indicating that no single study entirely accounted for the observed variability. One potential factor contributing to this heterogeneity is the finding that, in both Present studies a and b, patients with schizophrenia showed nominally lower C compared to those with bipolar disorder, unlike the other studies. This may be partly due to the extremely small sample sizes of the bipolar disorder groups (*n* = 26 and *n* = 14), which could have led to unstable mean estimates and exaggerated group differences. Such sample size imbalance likely contributed to the observed heterogeneity in C across studies.

This study has several limitations. Most of the participants were assessed via the NEO-FFI, NEO-PI-R, or NEO-related inventory and were diagnosed according to international criteria, namely, the DSM-III-R, DSM-IV, DSM-5 or ICD-10 (Table 1). Therefore, differences in assessment tools and diagnostic criteria may have affected our findings. There was also a lack of information for some study participants in each group, such as acute/chronic phase, in/outpatient, AAO, DOI, and diagnostic criteria, which may have affected the results. In addition, we could not perform a meta-analysis of personality traits between specific subtypes of bipolar disorder (types I and II) and healthy controls because there was no information about specific subtypes in each study. Previous study has indicated that bipolar II disorder patients exhibited higher N and lower E than bipolar I disorder patients.^44^ Thus, our results regarding N and E might have been affected by the subtypes of bipolar disorder (types I and II). Since all personality assessments in this study were conducted after the onset of disorders, it is difficult to determine whether the observed personality traits reflect premorbid characteristics or are secondary to the disorder. Moreover, the dataset lacks stratification by disorder phase, which is particularly important given that personality assessments may be significantly influenced by acute symptomatology, such as active psychotic episodes. Therefore, future longitudinal studies that assess premorbid personality traits and incorporate phase-stratified analyses are needed to better distinguish inherent vulnerability factors from state-dependent or disorder-related changes.

In conclusion, our data suggest that patients with schizophrenia have a characteristic personality profile (lower E, O, and A) compared with those with bipolar disorder. These effect sizes were modest: E (*g* = −0.40), O (*g* = −0.22), and A (*g* = −0.24), which were intermediate between personality traits in schizophrenia patients compared with controls^18^ and personality traits in bipolar disorder patients compared with controls.^19^ These results suggest that although schizophrenia patients and bipolar patients display personality profiles similar to those of controls, schizophrenia patients have more pronounced personality profiles in terms of E, O and A than bipolar disorder patients do.

## Supplementary Material

Supporting_information_pyaf060

## Data Availability

The datasets generated and/or analyzed during the current study are available from the corresponding author upon reasonable request. The data are not publicly available because they contain information that could compromise research participant privacy/consent.
