# Supplementary material for: Five-factor personality traits in patients with schizophrenia and bipolar disorder: a systematic review and meta-analysis
Source: Int J Neuropsychopharmacol. 2025 Aug 8;28(9):pyaf060. doi: 10.1093/ijnp/pyaf060 (PMC12418928; doi:10.1093/ijnp/pyaf060)

**Supporting information**

**Supplementary Table 1:** Demographic characteristics of patients with SCZ and BD, Kanazawa and Gifu, Japan.

|  | Kanazawa | | | |  | Gifu | | | |
| --- | --- | --- | --- | --- | --- | --- | --- | --- | --- |
|  | SCZ | BD |  |  |  | SCZ | BD |  |  |
| Variables | (*n*=182) | (*n*=26) | *z or x^2^* | *p* value |  | (*n*=53) | (*n*=14) | *z or x^2^* | *p* value |
| Age (years) | 47.2 ± 13.5 | 50.5 ± 17.5 | 1.0 | 0.31 |  | 40.5 ± 17.6 | 53.9 ± 16.8 | 2.5 | **0.011** |
| Sex (male/female) | 85/97 | 11/15 | 0.2^a^ | 0.67 |  | 19/34 | 4/10 | 0.3^a^ | 0.61 |
| Education (years) | 12.5 ± 2.1 | 14.0 ± 3.5 | 2.2 | **0.030** |  | 12.7 ± 2.7 | 12.4 ± 1.8 | -0.7 | 0.46 |
| Estimated premorbid IQ | 98.0 ± 10.6 | 101.5 ± 12.4 | 1.1 | 0.28 |  | 98.5 ± 10.1 | 101.3 ± 9.6 | 0.9 | 0.36 |
| CPZ-eq (mg/day) | 534.2 ± 495.0 | 123.4 ± 184.7 | -5.4 | **8.21×10^-8^** |  | 339.3 ± 385.2 | 207.3 ± 312.9 | -1.6 | 0.12 |
| BPD-eq (mg/day) | 1.1 ± 2.4 | 0 | -3.1 | **2.00×10^-3^** |  | 0.1 ± 0.6 | 0 | -0.9 | 0.37 |
| IMI-eq (mg/day) | 5.8 ± 28.7 | 33.4 ± 73.4 | 3.7 | **1.90×10^-4^** |  | 11.3 ± 44.6 | 5.4 ± 20.0 | -0.4 | 0.66 |
| Age at onset (years) | 26.9 ± 10.2 | 35.0 ± 17.1 | 2.3 | **0.024** |  | 29.2 ± 13.7 | 35.1 ± 16.5 | 1.3 | 0.19 |
| DOI (years) | 20.2 ± 13.1 | 15.5 ± 12.9 | -1.7 | 0.085 |  | 11.3 ± 11.1 | 18.7 ± 10.1 | 2.4 | **0.015** |
| PANSS positive symptoms | 15.7 ± 6.2 | - | - | - |  | 16.4 ± 5.6 | - | - | - |
| PANSS negative symptoms | 18.8 ± 6.6 | - | - | - |  | 16.1 ± 5.2 | - | - | - |
| HAMD-17 | - | 8.5 ± 6.1 | - | - |  | - | 9.3 ± 8.3 | - | - |
| YMRS | - | 1.2 ± 2.8 | - | - |  | - | 9.3 ± 9.4 | - | - |

SCZ, schizophrenia; BD, bipolar disorder; IQ, intelligence quotient; CPZ-eq, total antipsychotic dosage in chlorpromazine equivalents; BPD-eq, biperiden equivalents of total antiparkinsonian drugs; IMI-eq, imipramine equivalents of total antidepressants; DOI, duration of illness; PANSS, Positive and Negative Syndrome Scale; HAMD-17, 17-item Hamilton Rating Scale for Depression; YMRS, Young Mania Rating Scale. Complete demographic information was not obtained for all the subjects. The number of participants for which the estimated premorbid IQ was available was as follows: SCZ (Kanazawa), *n*=172; BD (Kanazawa), *n*=26; SCZ (Gifu), *n*=50; BD (Gifu), *n*=14. Number of participants for which the HAMD-17 was available: BD (Kanazawa), *n*=23; BD (Gifu), *n*=13. Number of participants for which the PANSS positive and negative symptoms were available: SCZ (Gifu), *n*=50. Number of participants for which the YMRS was available: BD (Gifu), *n*=13. The means ± SDs are shown. ^a^ *χ^2^* test. *P* values <0.05 are shown in boldface.

**Supplementary Figure 1**: Funnel plots for the meta-analyses of differences in each personality trait between patients with schizophrenia and those with bipolar disorder. Funnel plots of standard error by Hedges’ *g* are indicated. Each circle represents the result of an individual study. The diamond at the bottom of each figure represents the overall effect size (Hedges’ *g*).


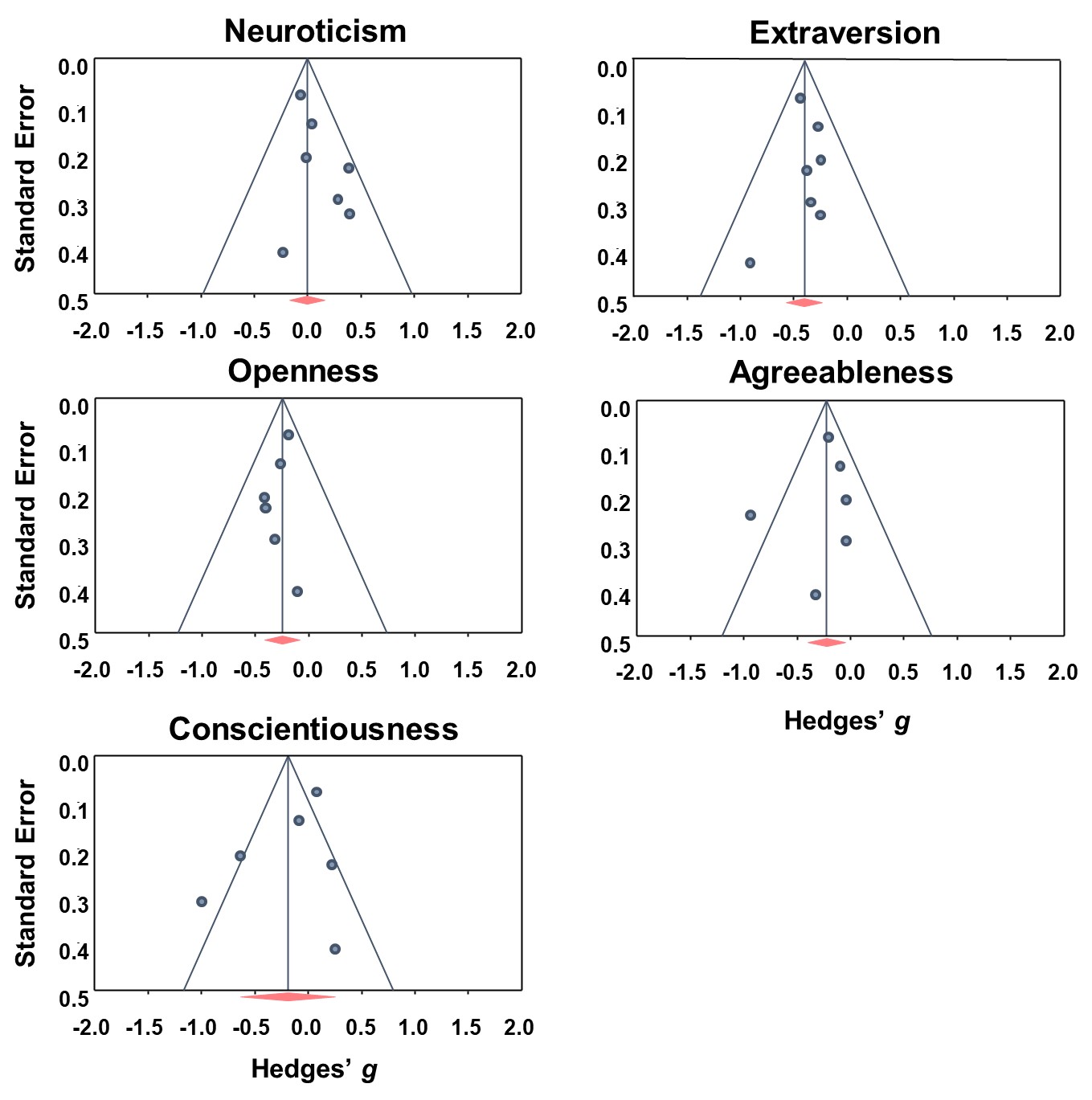

Supplement: Supporting_information_pyaf060 [file supporting_information_pyaf060.docx]
